# Supplementary material for: Simultaneous and stoichiometric purification of hundreds of oligonucleotides
Source: Nat Commun. 2018 Jun 25;9:2467. doi: 10.1038/s41467-018-04870-w (PMC6018234; doi:10.1038/s41467-018-04870-w)
Supplement: Supplementary file 1 — Supplementary Information [file 41467_2018_4870_MOESM1_ESM.pdf]

Simultaneous and Stoichiometric Purification of Hundreds of Oligonucleotides

Alessandro Pinto,[1] Sherry X. Chen,[1] and David Yu Zhang[1]
[1]*Department of Bioengineering, Rice University, Houston, TX*

Supplementary Figures	1
Supplementary Tables	14
Supplementary Notes	15
Supplementary Discussion	18
Supplementary Reference	20

Supplementary Figures

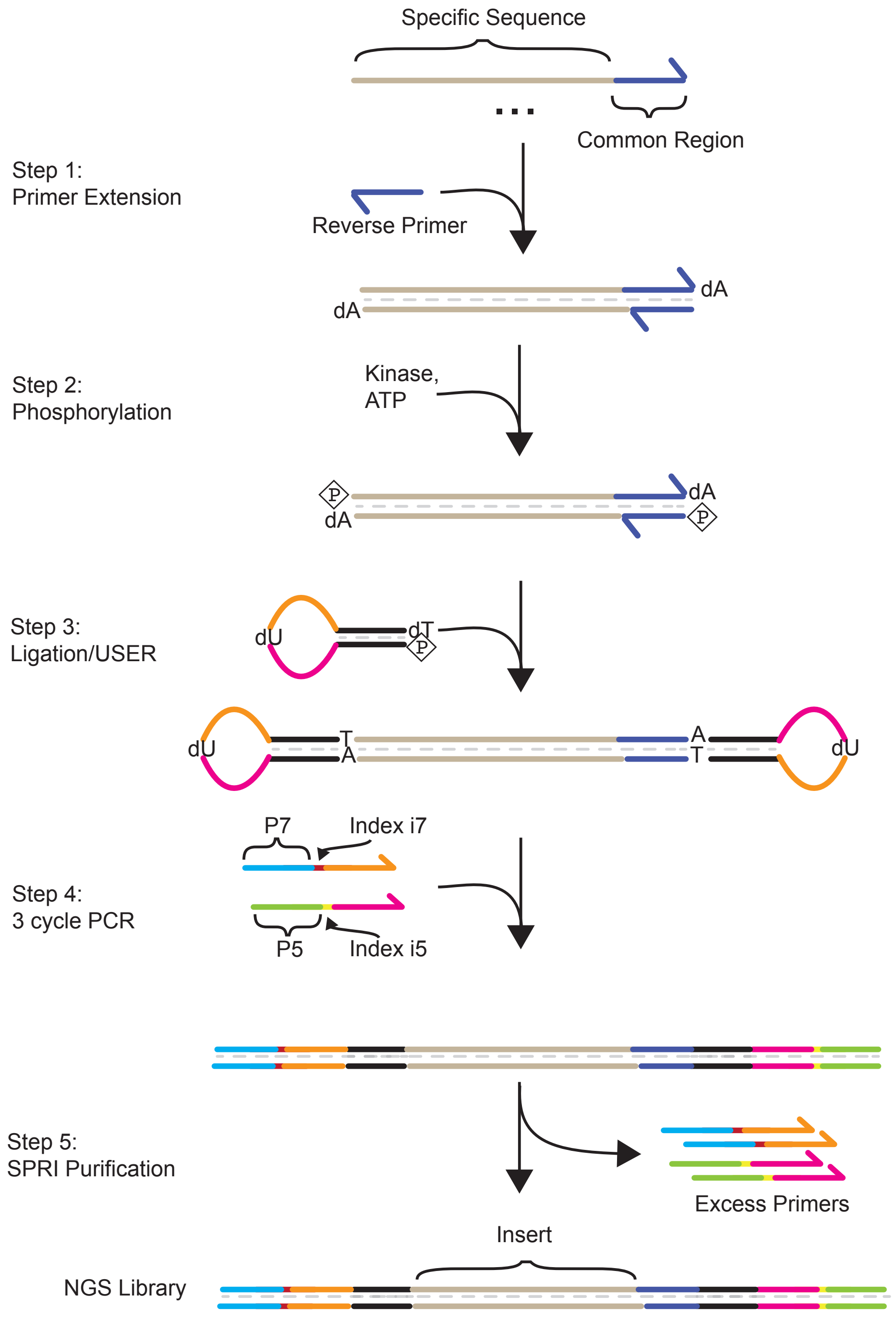

Supplementary Figure 1: Default NGS library preparation method for analyzing oligo purity and concentrations. The i7 indexes used were N701 through N712, and the i5 indexes used were E502 through E505.

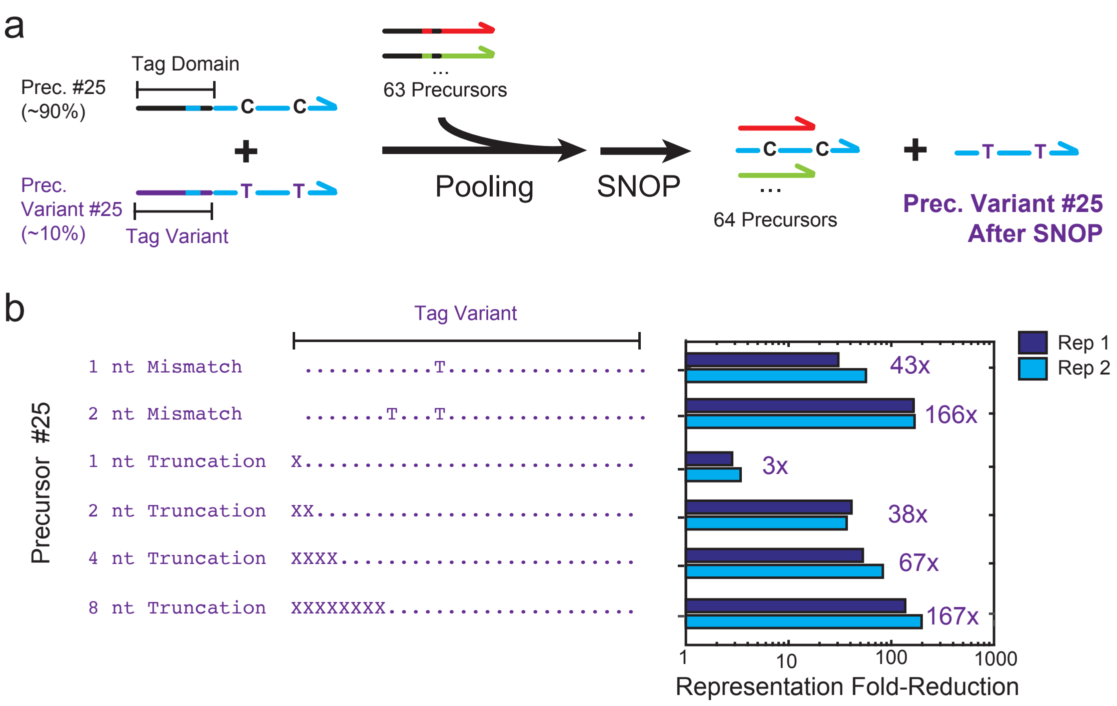

Supplementary Figure 2: Layout and results of our experiments on the effect of imperfect tag sequences on SNOP capture yield, which indirectly affects the purity of SNOP products. **(a)** Two versions of precursor #25 were created and mixed together in each experiment – an oligo with the intended tag sequence and intended product or "cargo" sequence (90%), and an oligo with a variant tag sequence and a variant product sequence (10%). NGS analysis of the product sequence reveal the relative capture yields of precursors with different tag sequences. **(b)** Tag variants and their effects on capture yield. 'X' indicates an intentionally truncated nucleotide in the tag, and '.' indicates the same sequence as the perfect tag. Two independent replicate experiments were performed for reproducibility analysis. Modified sequences are specified in Supplementary Table 1.

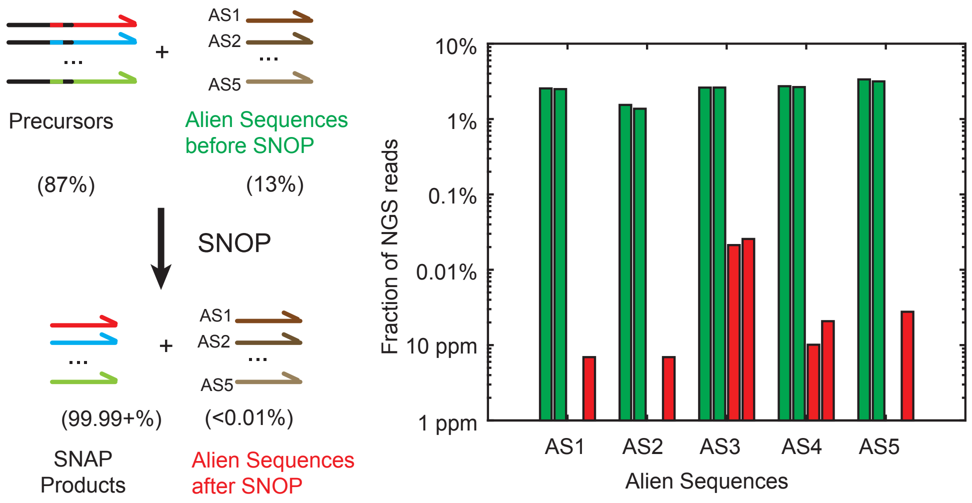

Supplementary Figure 3: Characterization of nonspecific capture/binding during the SNOP process due to magnetic bead and plasticware adsorption. Five (5) different "alien" sequences were designed with no tag sequence, and introduced into the precursor mixture for 64-plex SNOP. The alien sequence collectively comprise 13% of the total number of molecules in the pre-SNOP mixture. After SNOP, less than 0.01% of all molecules correspond to the alien sequences, based on NGS analysis. Shown here in the bar graph are results from 2 independent NGS runs. Thus, nonspecific binding should not be a significant contributor to remaining impurities in SNOP products. Alien sequences are specified in Supplementary Table 1.

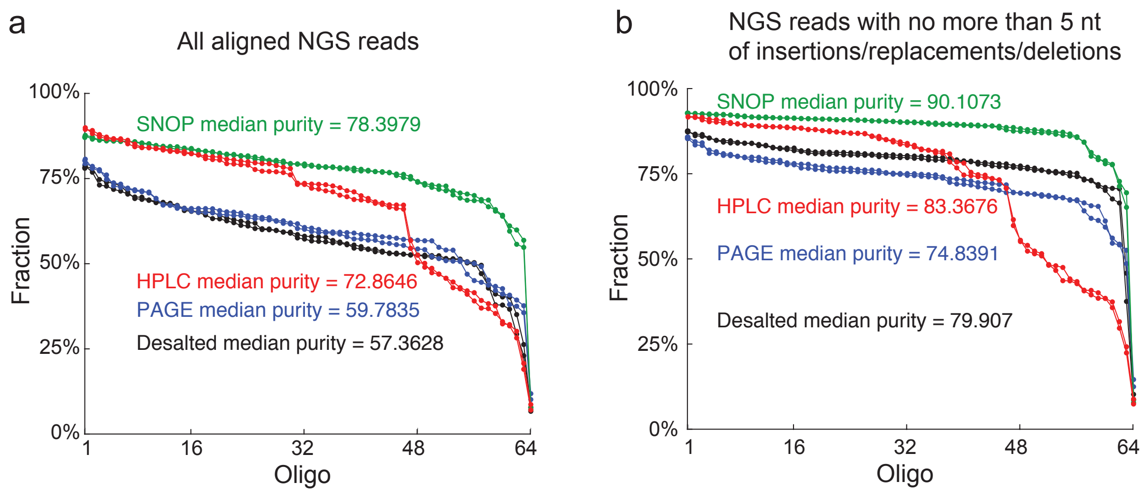

Supplementary Figure 4: Implied oligo purity from NGS reads (64-plex SNOP). **(a)** Oligo purity distribution using all aligned NGS reads. Recall that purity is defined as the number of NGS reads that perfectly align to the oligo, divided by the number of NGS reads that align based on Bowtie2 [5]. **(b)** Implied oligo purities based on NGS reads that align to their respective oligo sequences with no more than 5 nucleotide errors (insertions, deletions, or replacements). This analysis likely removes most if not all library preparation artifacts (e.g. adaptor-oligo dimers), but also removes some molecules with real truncations or deletions greater than 5 nt. Consequently, the true purity distribution likely lies between the two distributions shown in panels (a) and (b).

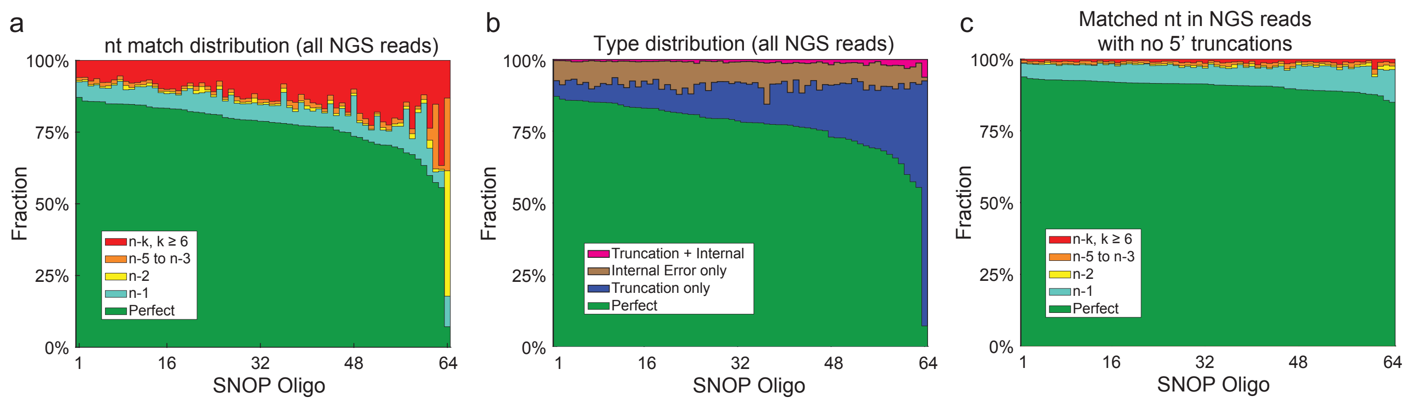

Supplementary Figure 5: Analysis of NGS reads for 64-plex SNOP. **(a)** Distribution of NGS reads, based on the number of nucleotides matched to the reference oligo sequences. The three most prevalent classes of species are perfect matches (green), single-base truncations and deletions (cyan), and gross errors (6 or more unmatched nucleotides, red). **(b)** Distribution of NGS reads with truncations, internal errors, and both. **(c)** Analysis of NGS reads with no 5′ truncations, representing the ideal outcome if SNOP is capable of removing all molecules with imperfect tag sequences.

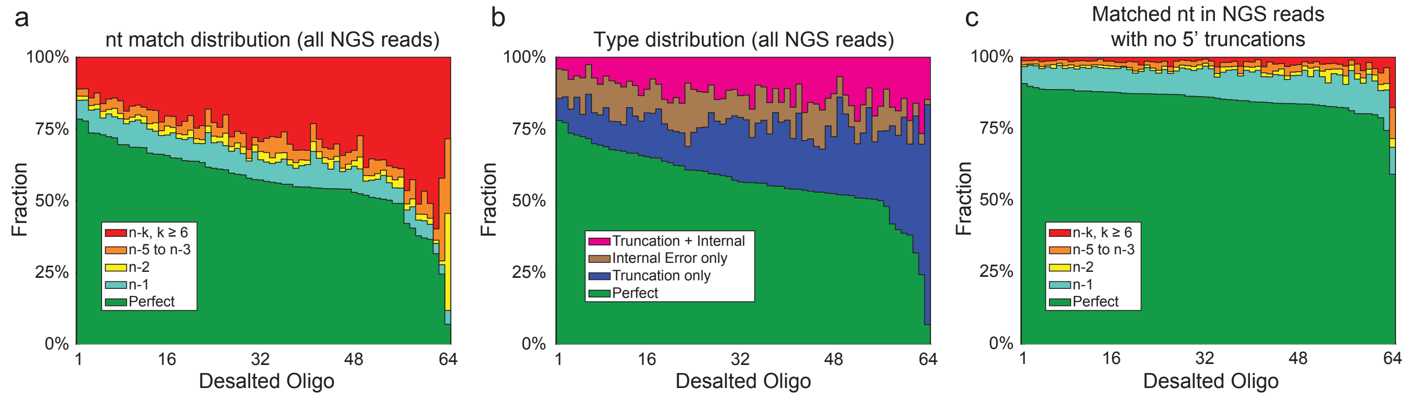

Supplementary Figure 6: Analysis of NGS reads for 64 desalted oligos with the same sequences as the 64-plex SNOP products. **(a)** Distribution of NGS reads, based on the number of nucleotides matched to the reference oligo sequences. **(b)** Distribution of NGS reads with truncations, internal errors, and both. **(c)** Analysis of NGS reads with no 5′ truncations.

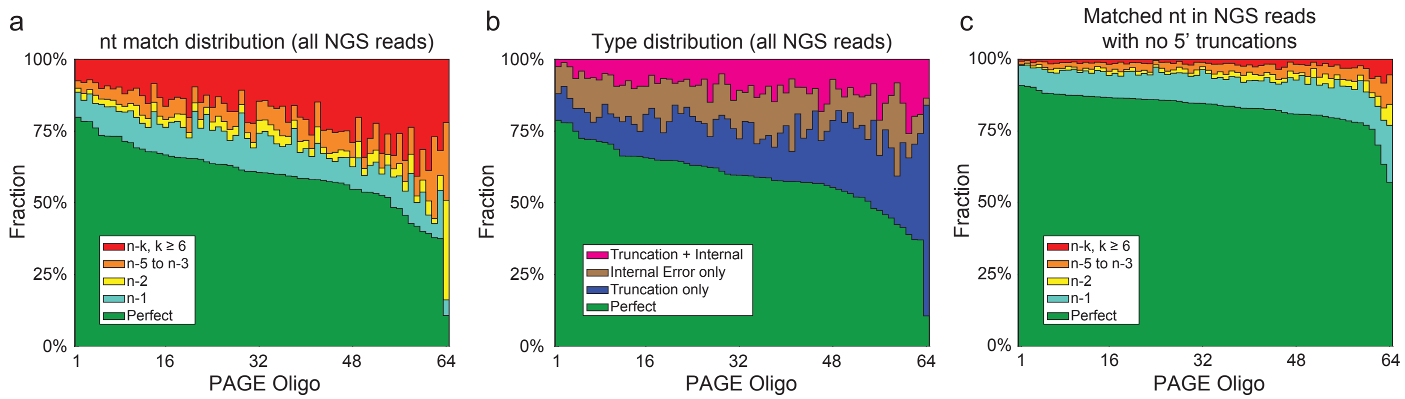

Supplementary Figure 7: Analysis of NGS reads for 64 commercially supplied PAGE-purified oligos with the same sequences as the 64-plex SNOP products. **(a)** Distribution of NGS reads, based on the number of nucleotides matched to the reference oligo sequences. **(b)** Distribution of NGS reads with truncations, internal errors, and both. **(c)** Analysis of NGS reads with no 5′ truncations.

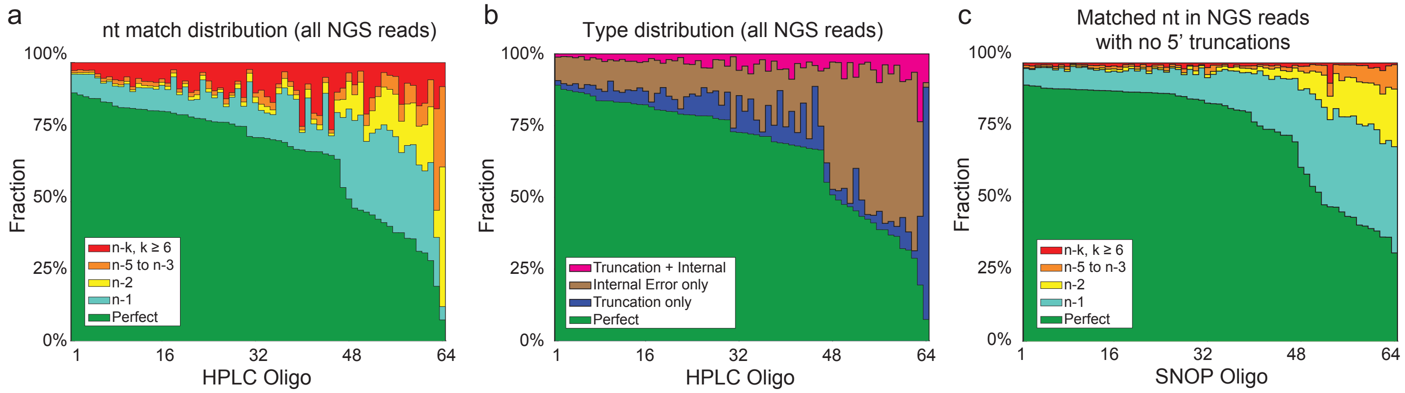

Supplementary Figure 8: Analysis of NGS reads for 64 commercially supplied HPLC-purified oligos with the same sequences as the 64-plex SNOP products. **(a)** Distribution of NGS reads, based on the number of nucleotides matched to the reference oligo sequences. **(b)** Distribution of NGS reads with truncations, internal errors, and both. **(c)** Analysis of NGS reads with no 5′ truncations.

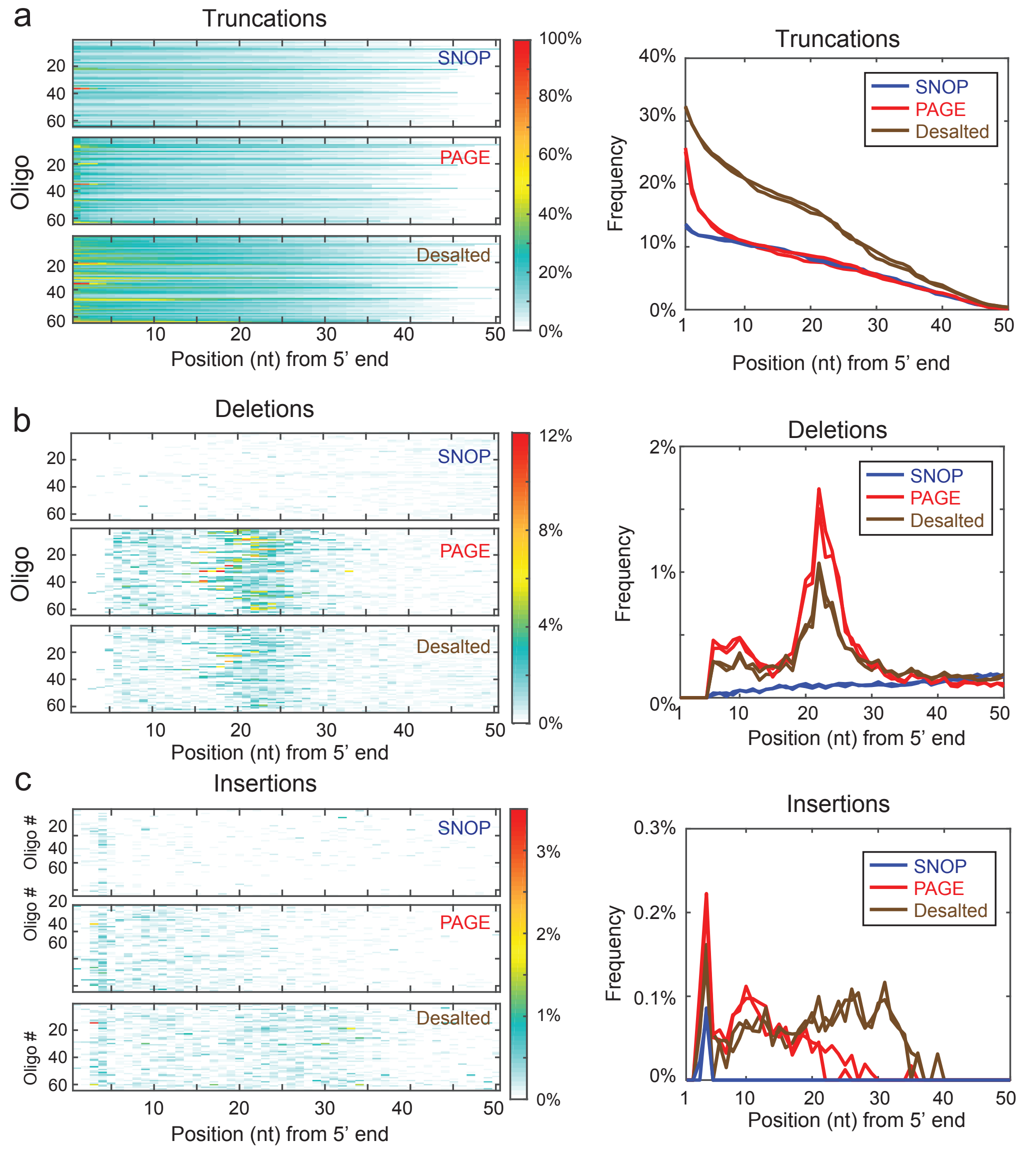

Supplementary Figure 9: Positional distribution of truncations, deletions, and insertions for the oligos used in 64-plex SNOP. Because the reverse primer binds to positions 50 through 70, errors in those positions likely correspond to primer synthesis errors, rather than precursor synthesis errors, and consequently are not shown here. **(a)** Truncation distribution. **(b)** Internal deletion distribution. **(c)** Insertion distribution.

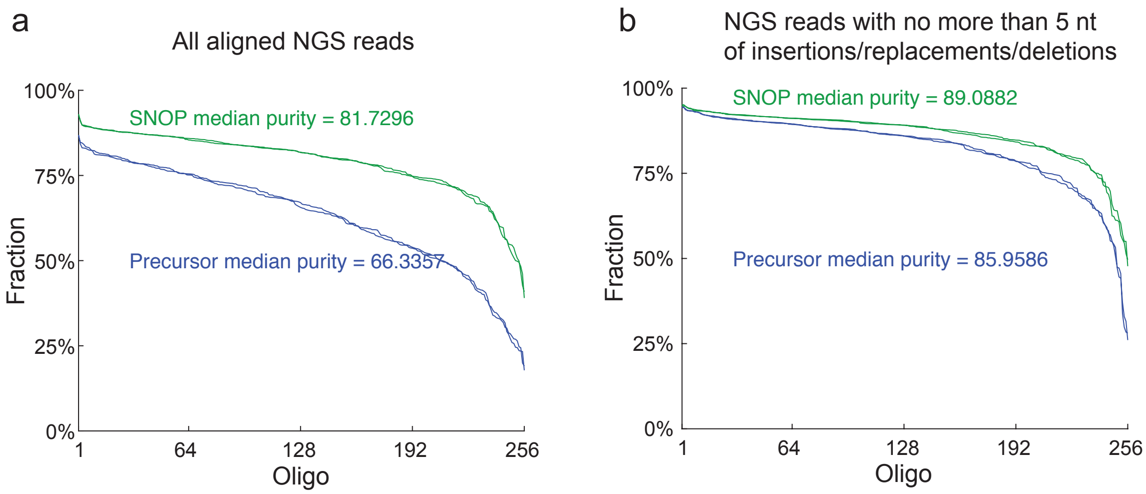

Supplementary Figure 10: Implied oligo purity from NGS reads (256-plex SNOP). **(a)** Oligo purity distribution using all aligned NGS reads. **(b)** Implied oligo purities based on NGS reads that align to their respective oligo sequences with no more than 5 nucleotide errors (insertions, deletions, or replacements). The true purity distribution likely lies between the two distributions shown in panels (a) and (b).

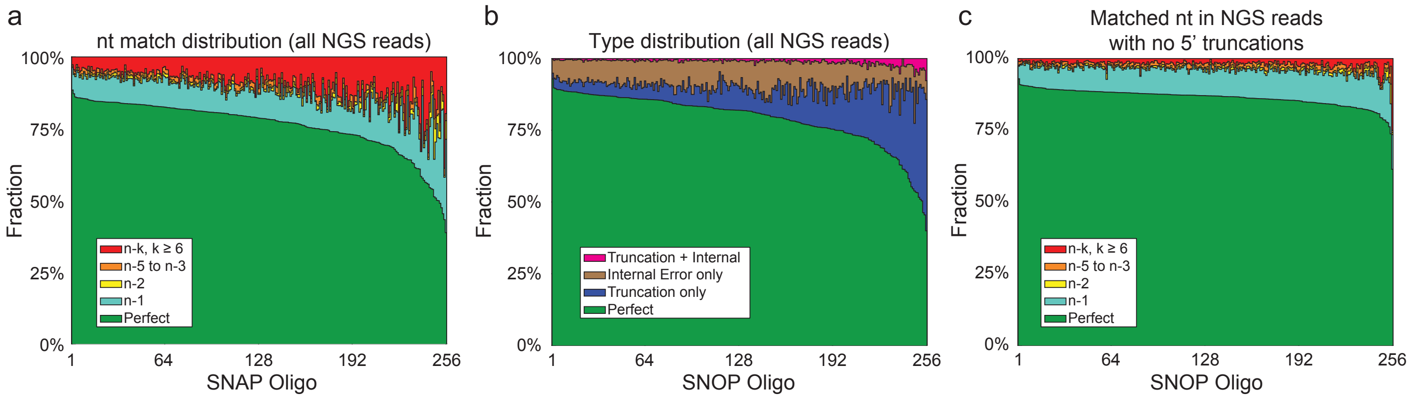

Supplementary Figure 11: Analysis of NGS reads for 256-plex SNOP products. **(a)** Distribution of NGS reads, based on the number of nucleotides matched to the reference oligo sequences. **(b)** Distribution of NGS reads with truncations, internal errors, and both. **(c)** Analysis of NGS reads with no 5′ truncations.

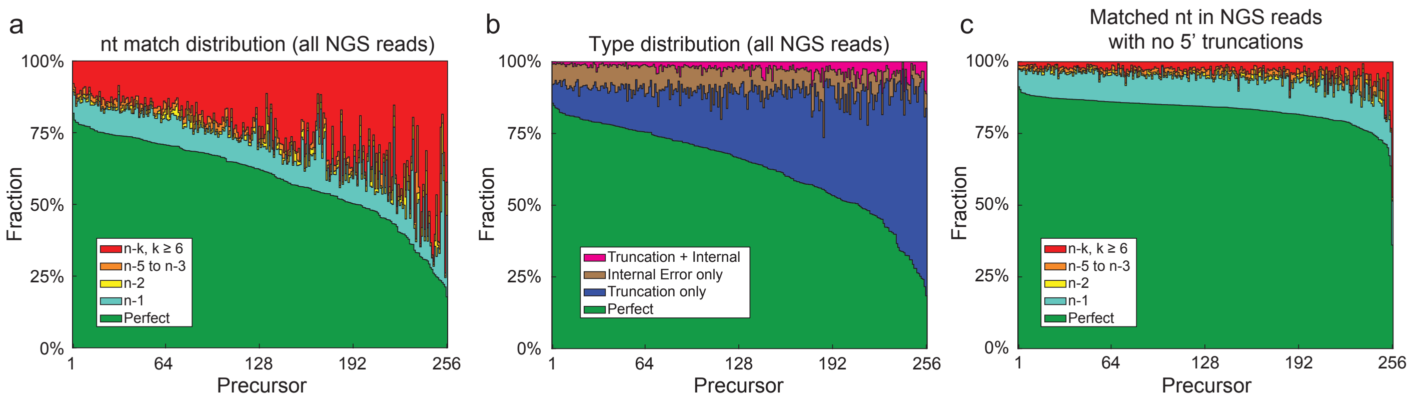

Supplementary Figure 12: Analysis of NGS reads for the 256 oligos used as precursors for the 256-plex SNOP. **(a)** Distribution of NGS reads, based on the number of nucleotides matched to the reference oligo sequences. **(b)** Distribution of NGS reads with truncations, internal errors, and both. **(c)** Analysis of NGS reads with no 5′ truncations.

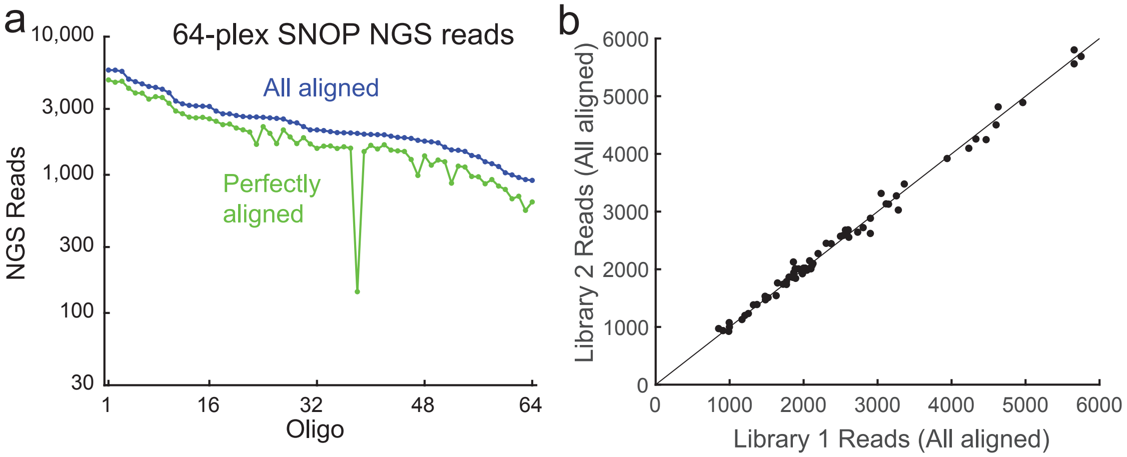

Supplementary Figure 13: NGS read distribution for 64-plex SNOP products. **(a)** Distribution of NGS reads aligned to each SNOP product. **(b)** Reproducibility of NGS reads across two identically prepared SNOP product libraries.

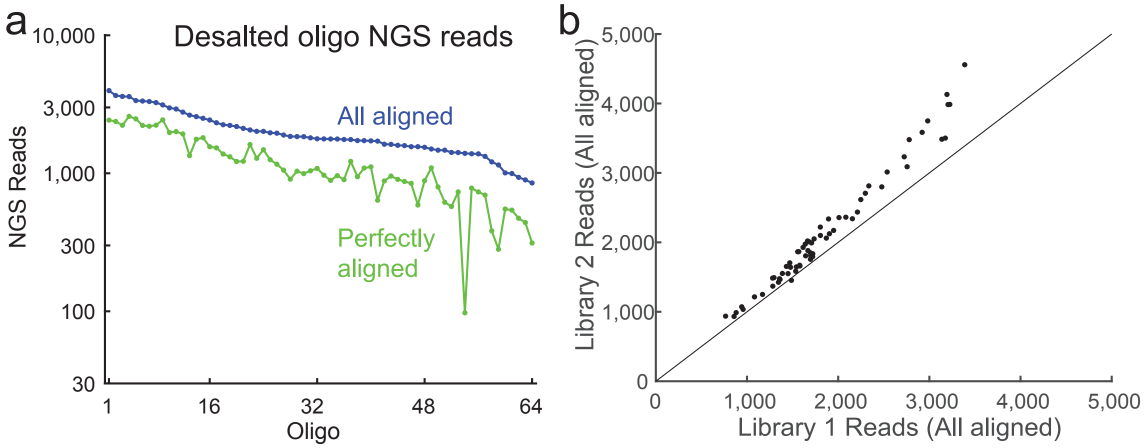

Supplementary Figure 14: NGS read distribution for the 64 desalted oligos that serve as normalization reference for concentration determination. **(a)** Distribution of NGS reads aligned to each oligo. **(b)** Reproducibility of NGS reads across two identically prepared desalted oligo libraries.

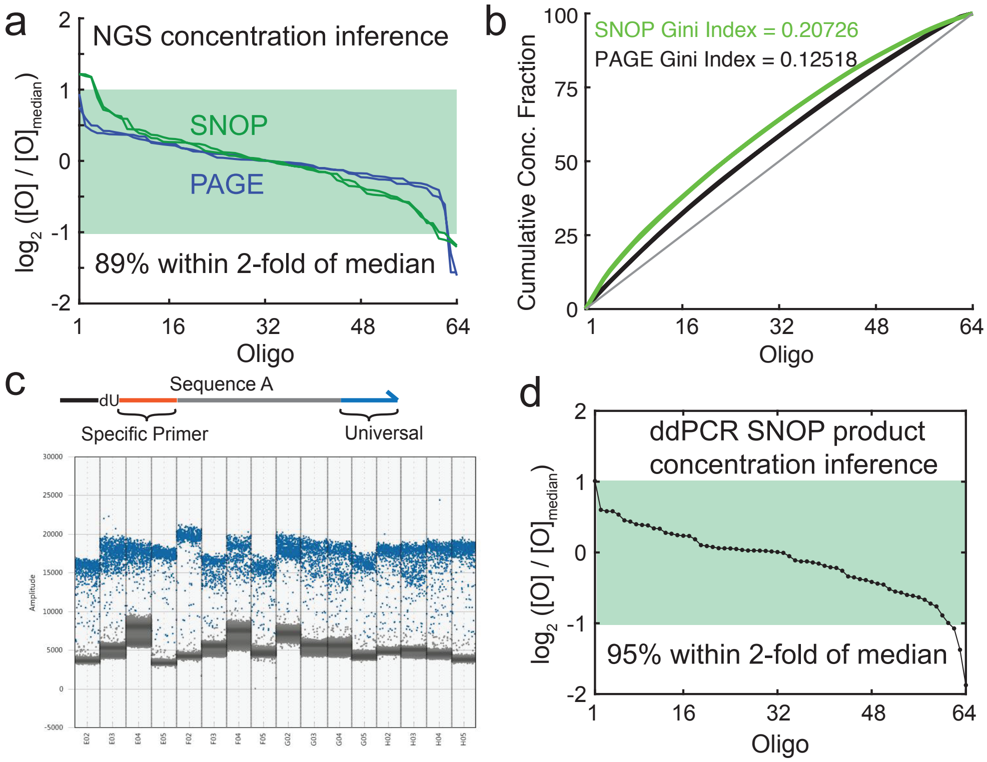

Supplementary Figure 15: 64-plex SNOP relative concentrations **(a)** Relative concentration distribution inferred via normalization to aligned reads in the desalted library. All oligos in the PAGE library at nominally the same concentration, and differences in relative concentration likely represent a combination of pipetting/dilution errors and NGS errors. **(b)** Cumulative distribution plot of relative concentrations, and calculated Gini coefficients based on NGS concentration inferences. **(c)** Sample digital PCR run results for 16 SNOP products. The Y-axis shows fluorescence and the X-axis shows the different droplets. Solid vertical lines separate different samples. **(d)** Relative concentrations inferred through digital droplet PCR (Biorad QX200). The distribution is highly similar to the NGS inference distribution.

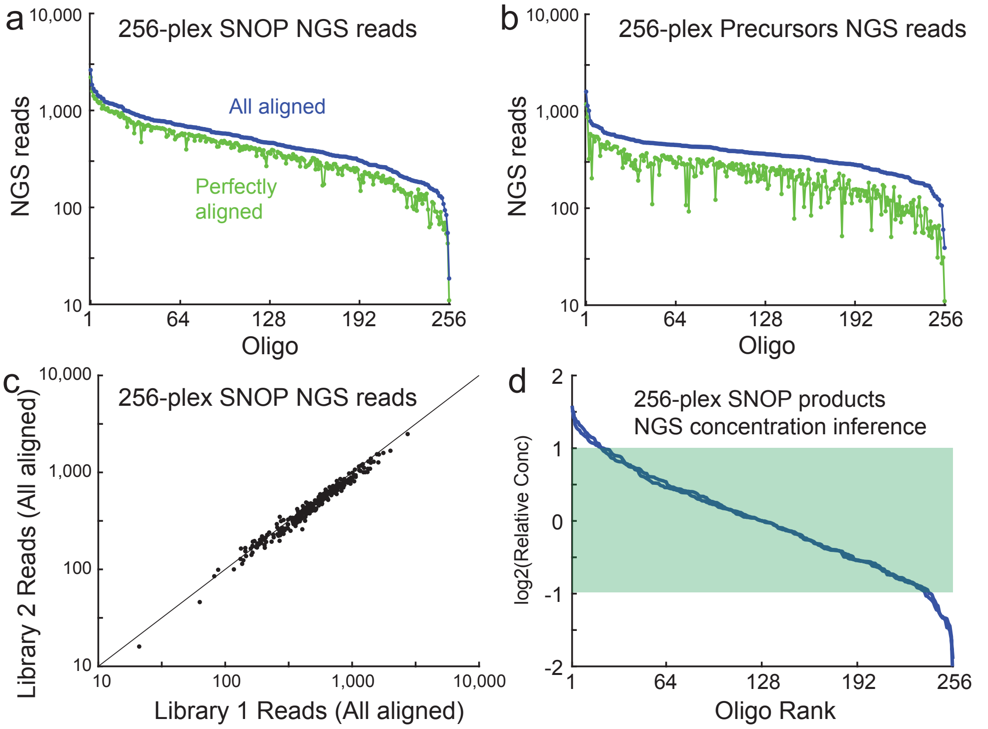

Supplementary Figure 16: NGS read distribution for 256-plex SNOP products. **(a)** Distribution of NGS reads aligned to each SNOP product. **(b)** Reproducibility of NGS reads across two identically prepared SNOP product libraries.

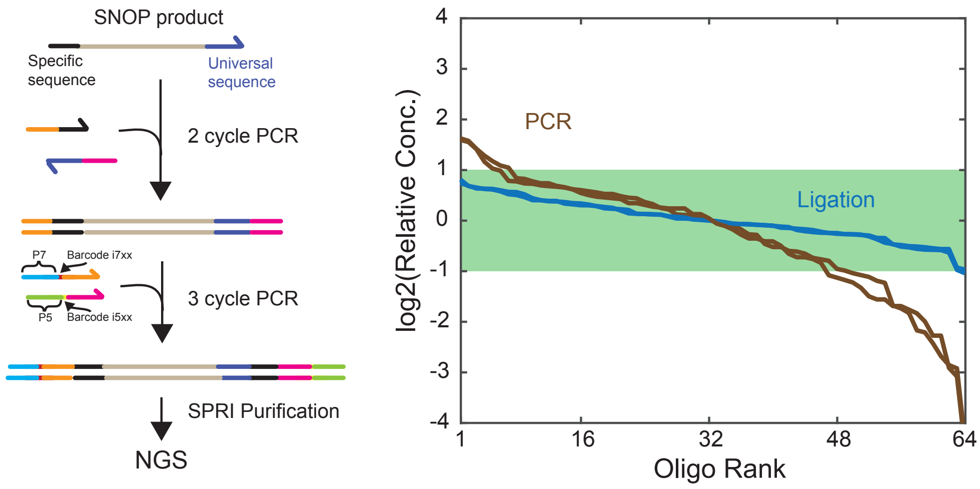

Supplementary Figure 17: Inferred relative concentrations for 64-plex SNOP using an alternative PCR-based protocol for appending sequencing primer sequences. There is significantly higher variation in the concentrations of the different oligo species, implying that PCR-based library preparation introduces higher concentration bias than our default ligation protocol.

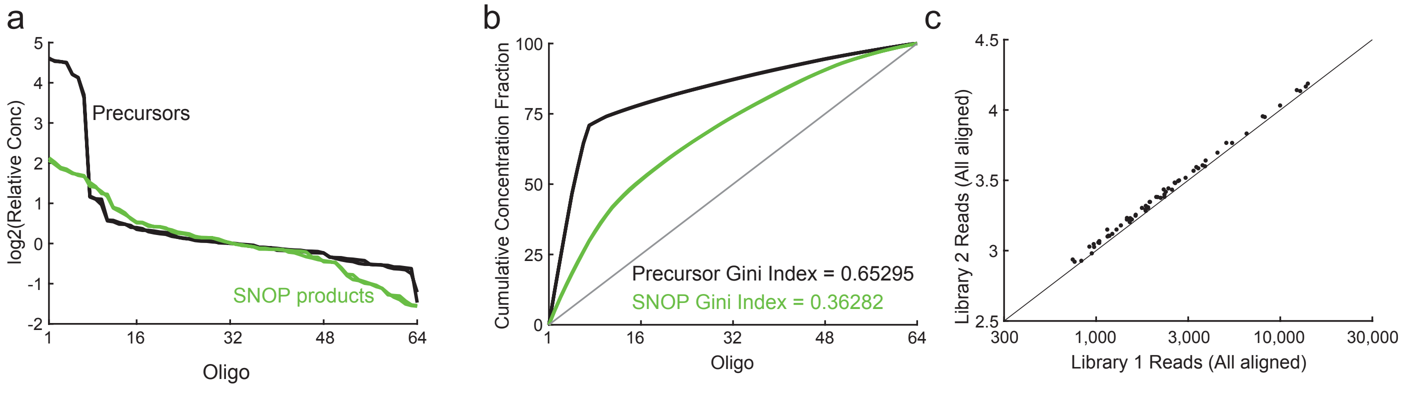

Supplementary Figure 18: NGS concentration analysis of SNOP robustness to precursor concentrations. 7 of the 64 precursors were introduced at 25-fold higher concentration than other precursors. **(a)** Inferred concentrations of precursors and SNOP products. **(b)** Cumulative concentration distribution and calculated Gini indices. **(c)** Reproducibility of aligned SNOP product NGS reads across two independent runs.

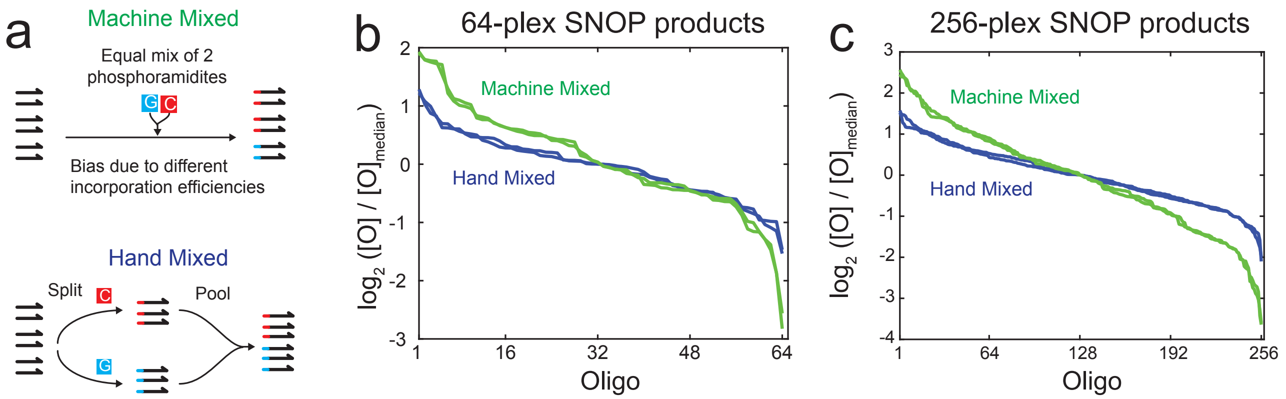

Supplementary Figure 19: Effects of probe instance concentration bias on SNOP product concentrations. **(a)** Two different options for synthesis of the randomer probe. The hand-mixed "split-pool" synthesis is expected to provide better uniformity of capture probe instances. **(b)** Comparison of relative concentration distribution on 64-plex SNOP using capture probe synthesized via hand mixed protocol vs. machine mixed protocol. **(c)** Comparison of relative concentration distribution for 256-plex SNOP.

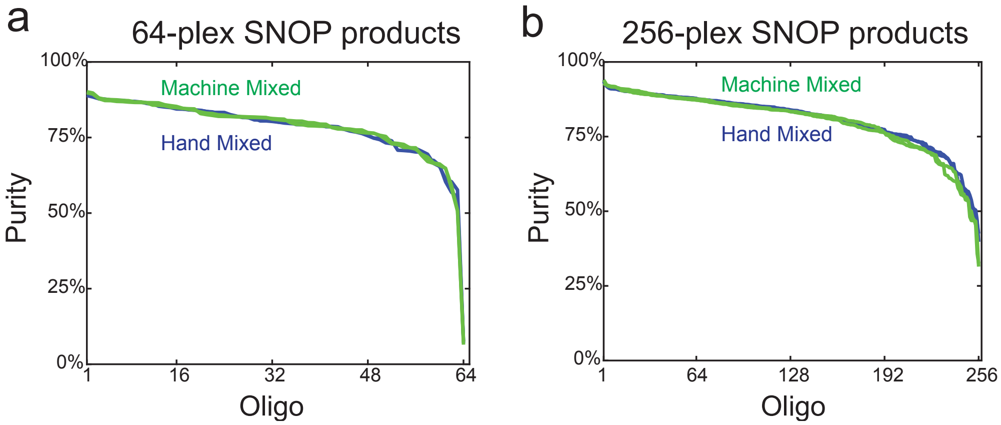

Supplementary Figure 20: Effects of probe instance concentration bias on SNOP product purities. The two different capture probe syntheses did not appear to yield significant differences in purity for either **(a)** 64-plex SNOP or **(b)** 256-plex SNOP.

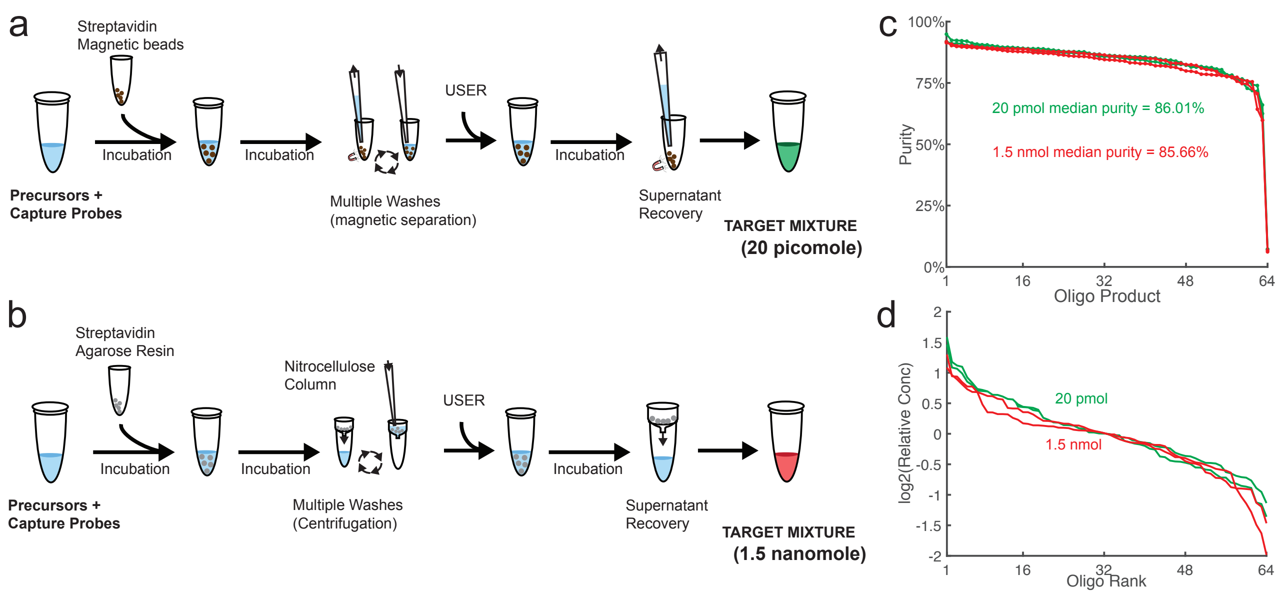

Supplementary Figure 21: Comparison of SNOP at different scales. **(a)** Original SNOP workflow using magnetic beads, yielding 20 picomole oligo products. **(b)** Scaled-up SNOP workflow using agarose resin, yielding 1.5 nanomole oligo pools. **(c)** Observed purity distribution for 64-plex SNOP using the 2 different workflows. **(d)** Observed stoichiometry distribution for 64-plex SNOP using the 2 different workflows.

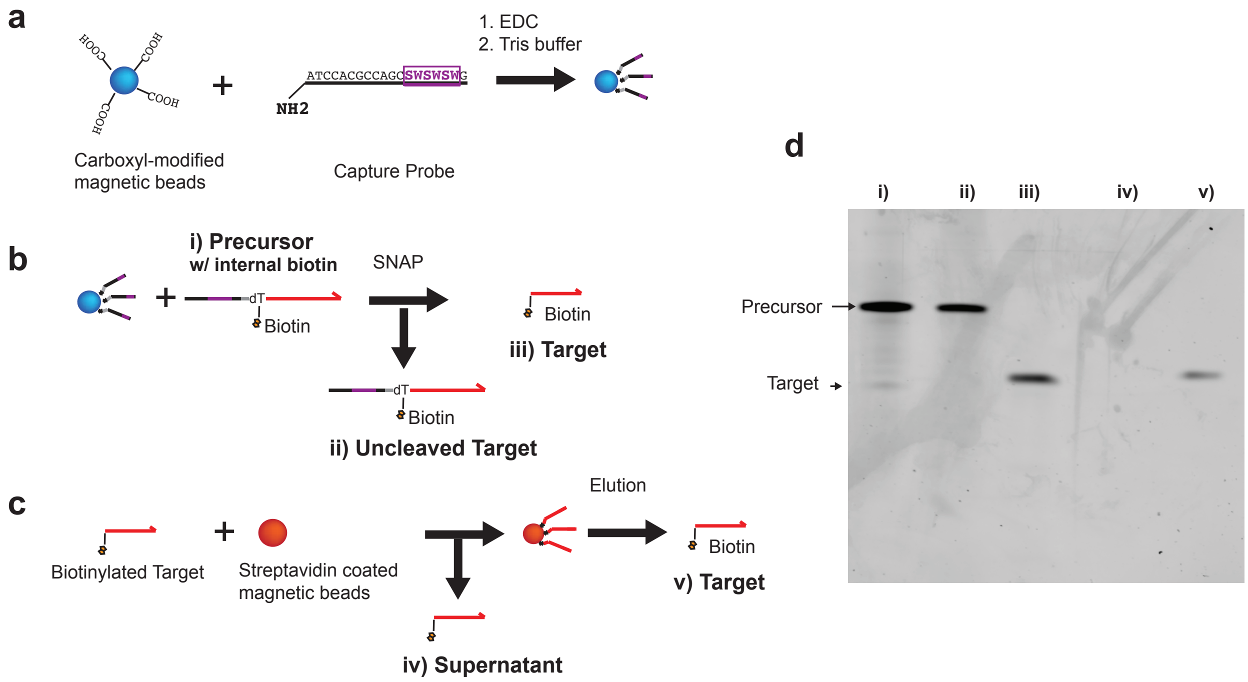

Supplementary Figure 22: Modified workflow for applying SNOP to biotinylated oligos. **(a)** Amino-coupling strategy used to immobilize the capture probe onto the carboxyl-functionalized magnetic beads. **(b)** Precursors bear an internal biotinylated thymine to the 3' of the tag sequence. **(c)** Demonstration of active biotin-functionalization on SNOP products. **(d)** Denaturing polyacrylamide gel electrophoresis (PAGE) stained with SYBR gold. Lanes correspond to the species described in panels (b) and (c). There is significant products in (v) but not in (iv), indicating that the majority of SNOP products maintain active biotin functionalization. Sequences are specified in Supplementary Table 1.

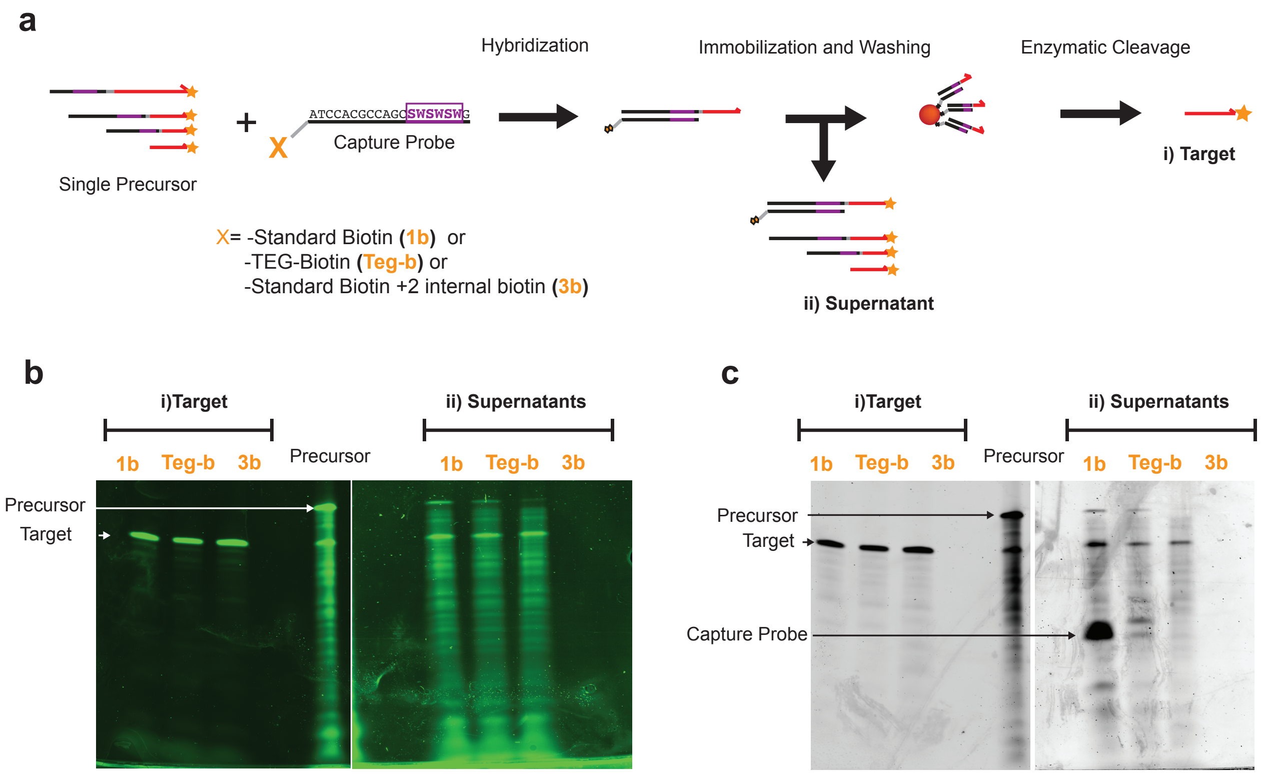

Supplementary Figure 23: Effects of different Biotin functionalizations on SNOP. **(a)** Workflow. **(b)** Fluorescent gel electrophoresis of SNOP products using different biotin functionalizations for the capture probe. **(c)** SYBR-Gold stained gel electrophoresis of SNOP products using different biotin functionalizations for the capture probe.

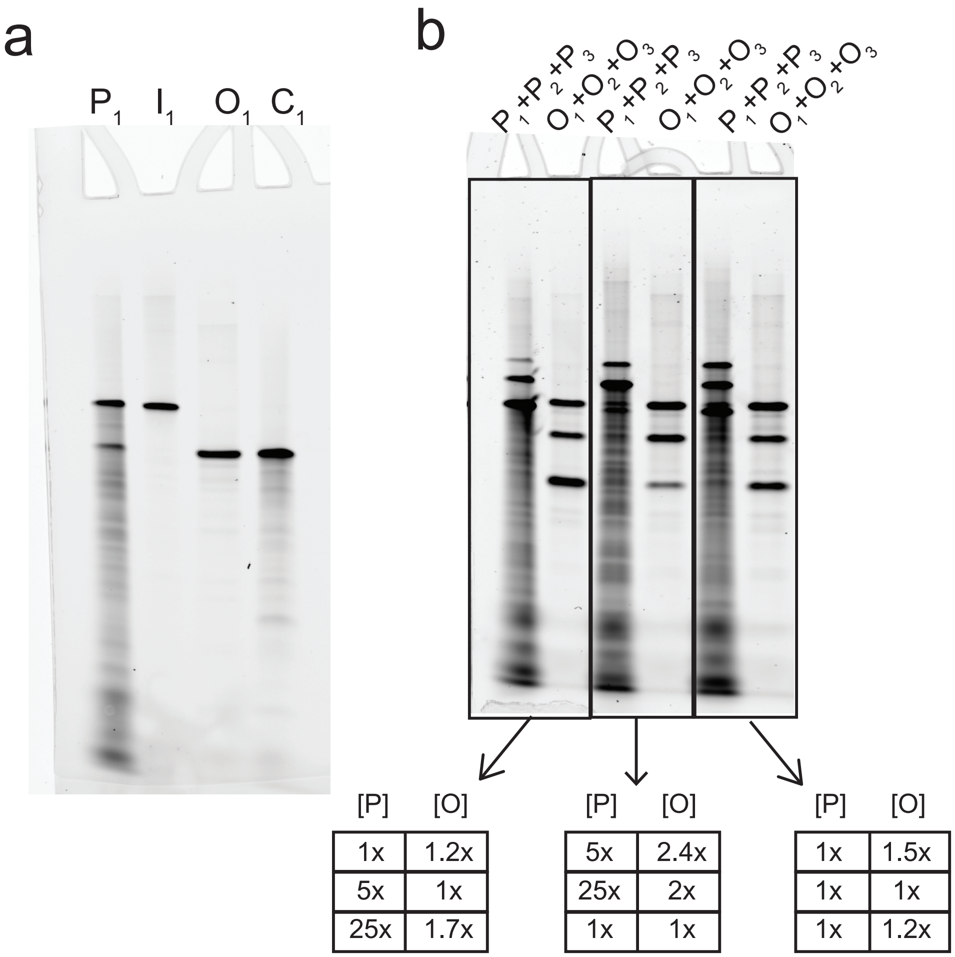

Supplementary Figure 24: Uncropped gels presented in the main text, Fig.2. **(a)** See description of Fig.2b.**(b)**See description of Fig.2e.

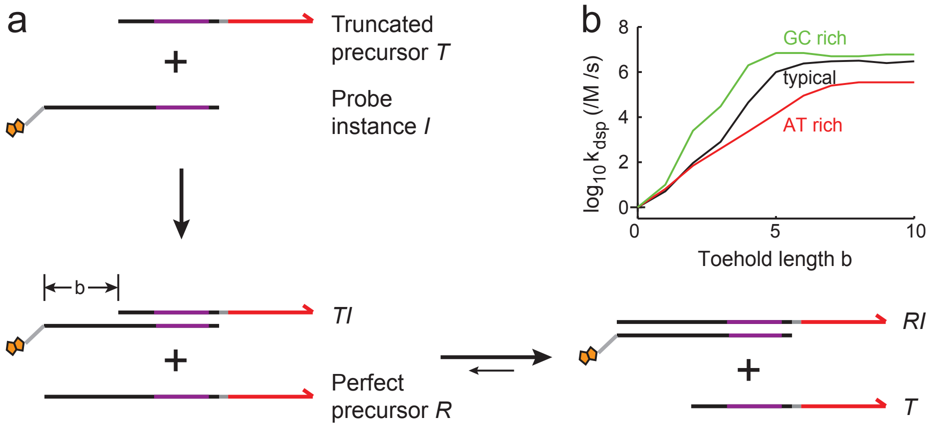

Supplementary Figure 25: Rapid displacement of truncated precursor T by perfect precursor R. **(a)** The number of bases b truncated in T serves as the toehold for the strand displacement reaction. **(b)** Dependence of rate constant of displacement k_{dsp} on b. Data previously published in Supplementary Reference [4].

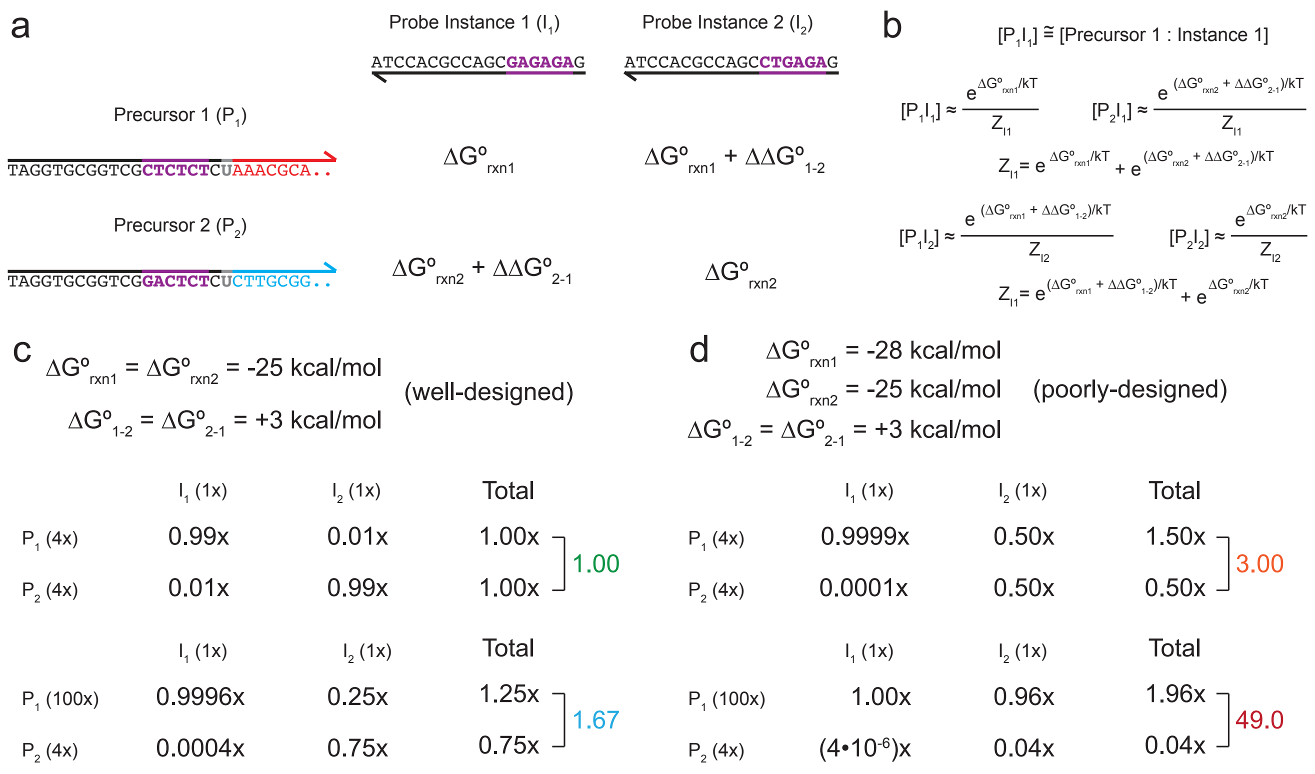

Supplementary Figure 26: Impact of reaction standard free energy on product stoichiometry. **(a)** Potential hybridization reactions with 2 precursors and 2 probe instances. **(b)** Approximation of equilibrium captured precursor concentration. **(c)** Quantitative example of product stoichiometry using a well-designed SNOP system. **(d)** Quantitative example of product stoichiometry using a poorly designed SNOP system.

Supplementary Table

Supplementary Table 1: Sequences used for Supplementary Figures 2,3 and 22. A comprehensive list of sequences and primers used in this work is given in the Supplementary Data File.

ID	Sequence	Provider	Type	Purification	Experiment
Pre_64plex_25_1M	TAGGTGCGGTCTCATGTGTCTCACTGUGTTCGGTTCATCCCGCAGCGCCAGTTCTGCTTATCGTGCCACAGTCTCGTACGGTTAAGAGCC	Sigma	1 Mutated nt	Desalt (none)	Supplementary Figure 2
Pre_64plex_25_2M	TAGGTGCTGTCTCATGTGTCTCACTGUGTTCGGTTCATCCCGCAGCGCCAGTTCTGCTTATCGTGTCACAGTCTCGTACGGTTAAGAGCC	Sigma	2 Mutated nt	Desalt (none)	Supplementary Figure 2
Pre_64plex_25_1T	AGGTGCGGTCGCATGTGTCTCACTGUGTTCGGTTCATCCCGCAGCGCCAGTTCTGCTTATCGTGTCACAGTCTCGTACGGTTAAGAGCC	Sigma	1 truncated nt	Desalt (none)	Supplementary Figure 2
Pre_64plex_25_2T	GGTGCGGTCGCATGTGTCTCACTGUGTTCGGTTCATCCCGCAGCGCCAGTTCTGCTTATCGTGTCACAGTCTCGTACGGTTAAGAGCC	Sigma	2 truncated nt	Desalt (none)	Supplementary Figure 2
Pre_64plex_25_4M	TGCGGTCGCATGTGTCTCACTGUGTTCGGTTCATCCCGCAGCGCCAGTTCTGCTTATCGTGTCACAGTCTCGTACGGTTAAGAGCC	Sigma	4 truncated nt	Desalt (none)	Supplementary Figure 2
Pre_64plex_25_8M	GTCGCATGTGTCTCACTGUGTTCGGTTCATCCCGCAGCGCCAGTTCTGCTTATCGTGTCACAGTCTCGTACGGTTAAGAGCC	Sigma	8 truncated nt	Desalt (none)	Supplementary Figure 2
Alien_Sequence_1	CGGATTGACCGCGACGTGAAAGCACGTCTCGAGTCAACCGTCTCGTACGGTTAAGAGCC	IDT	Alien_sequence	Desalt (none)	Supplementary Figure 3
Alien_Sequence_2	TACCAACTAAGTCTCGTACGGTTAAGAGCC	IDT	Alien_sequence	Desalt (none)	Supplementary Figure 3
Alien_Sequence_3	CGGATATCTCGTACGACGGAGTGACTAAGCACGTATAAACTAAGGTAGCTAGGGCCGGAAGTCTCGTACGGTTAAGAGCC	IDT	Alien_sequence	Desalt (none)	Supplementary Figure 3
Alien_Sequence_4	CAGGTATAGCATACAACATAGTCTCGTACGGTTAAGAGCC	IDT	Alien_sequence	Desalt (none)	Supplementary Figure 3
Alien_Sequence_5	CCCTTATGTTTCTCACGGGAGACATGTTTTAAAAAGTATCGTCTCGTACGGTTAAGAGCC	IDT	Alien_sequence	Desalt (none)	Supplementary Figure 3
Capture Probe Prototype	CAGWSWSWSACAGGCCGCAGGCGCTATTTTTTTTTTT/3AmMO/	Capture Probe	IDT	Desalt (none)	Supplementary Figure 22
Biotinylated Target	TAGCGCCTGCGGCCTGTGTGTGACTGU/iBiodT/CTTGCGGAACACGAATCGACCACTGACACAATTCGTAATCTCATTGCAAGCGTTT	Internal biotinylated target	IDT	HPLC	Supplementary Figure 22

Supplementary Notes

Supplementary Note 1: Single-plex SNOP Modeling. To mathematically explain the SNOP mechanism, we first consider a hypothetical, simplified 1-plex system, with precursor P and matching capture probe instance I. Precursor P can be further subdivided into four types of molecules: molecules with perfect sequence R, molecules with only sequence errors in the tag region T, molecules with only sequence errors in the product region D, and molecules with sequence errors in both the tag and product regions X. To first approximation, the reaction of each of the four types of species with I has the following thermodynamics:

$$R + I \rightarrow RI \qquad \Delta G^\circ_{\text{rxn}} \tag{1}$$

$$D + I \rightarrow DI \qquad \Delta G^\circ_{\text{rxn}} \tag{2}$$

$$T + I \rightarrow TI \qquad \Delta G^\circ_{\text{rxn}} + \Delta\Delta G^\circ \tag{3}$$

$$X + I \rightarrow XI \qquad \Delta G^\circ_{\text{rxn}} + \Delta\Delta G^\circ \tag{4}$$

Here, $\Delta G^\circ_{\text{rxn}}$ corresponds to the standard free energy of hybridization between perfect sequence and the capture probe. Its value can be calculated using software tools such as mFold [1] or Nupack [2] based on the sequences of R and I via $\Delta G^\circ_{\text{rxn}} = \Delta G^\circ(RI) - \Delta G^\circ(R) - \Delta G^\circ(I)$, where $\Delta G^\circ(RI)$ is the standard free energy of formation of the RI molecule. Each of the standard free energies of formation is subject to a roughly 1 kcal/mol standard error due to imperfections in the DNA hybridization thermodynamics models and parameters [3]. The true reaction standard free energies for Supplementary Equations (1) and (2) will differ slightly because R and D may have slightly different secondary structures, but except in extreme circumstances this difference is usually smaller than the standard error of ΔG° prediction.

The $\Delta\Delta G^\circ$ term in Supplementary Equations (3) and (4) corresponds to the relative thermodynamic penalties of due to imperfect hybridization between the tag and the probe for T and X. The value of $\Delta\Delta G^\circ$ will vary based on the number and identities of errors in the tag regions; for a single nucleotide truncation, $\Delta\Delta G^\circ$ ranges between 0.5 kcal/mol and 3 kcal/mol at the typical SNOP hybridization temperature of 60 °C.

Supplementary Note 2: Kinetic vs. Thermodynamic Regime. The above reactions can result in very different purities of SNOP products, based on whether the reactions are in a kinetic or a thermodynamic regime. In the kinetic regime, probe binding is irreversible, and whichever molecule first encounters a probe instance is captured. In this case, SNOP has no purification effect; both the initial and final purities are

$$\frac{R + T}{R + D + T + X} \tag{5}$$

In the thermodynamic regime, the binding efficiencies of R, D, T, and X are governed only by the reaction standard free energies. Assuming $\Delta\Delta G^\circ = 3$ kcal/mol (for a typical 2 nt truncation), T and X bind roughly 100-fold less efficiently than R and D. The final purity thus can be approximated as

$$\frac{R + 0.01 \cdot T}{R + D + 0.01 \cdot T + 0.01 \cdot X} \tag{6}$$

When synthesis errors are correlated, $\frac{X}{T+X}$ is greater than $\frac{D}{R+D}$, and the final purity will be greater than the initial purity.

The reason this reaction system is closer to the thermodynamic regime than to the kinetic regime is because the displacement of T and X species by R and D can be quite fast. The truncated bases essentially serve as a "toehold" for the displacement reaction (Supplementary Figure 24a); our previous studies of toehold-mediated strand displacement shows a strong exponential dependence of toehold length (Supplementary Figure 24b) [4], with rate constant kinetics saturating at the speed of hybridization for a toehold length of 6 nt for typical sequences.

For the standard SNOP hybridization time (2 hr) and precursor concentrations (100 nM each precursor), we expect that a strand displacement rate constant of

$$k_{\text{dsp}} \geq 1.4 \cdot 10^3 \text{ M}^{-1} \text{ s}^{-1} \tag{7}$$

is sufficient to for the displacement reaction to reach more than 1 half-life. This corresponds to a 3 nt toehold, so precursors with a 5 $'$ tag truncation of 3 nt or more likely are in the thermodynamic regime, while precursors with a 1 nt and 2 nt truncation are in the kinetic regime. In the limit of complete equilibrium, we would expect SNOP to achieve even higher purities via better separation of molecules with perfect tags vs. molecules with 1 or 2 nt

truncations. However, even with the current experimental restrictions, we observe significantly higher purities for SNOP products than for precursors.

Supplementary Note 3: Multiplex SNOP product stoichiometries. Next, to consider the stoichiometry of multiplex SNOP products, we consider a hypothetical 2-plex system, with precursors P_1 and P_2, and matching capture probe instances I_1 and I_2. The system can be modeled with the following four reactions:

$$P_1 + I_1 \rightarrow P_1 I_1 \qquad \Delta G^\circ_{\text{rxn1}} \tag{8}$$

$$P_2 + I_2 \rightarrow P_2 I_2 \qquad \Delta G^\circ_{\text{rxn2}} \tag{9}$$

$$P_1 + I_2 \rightarrow P_1 I_2 \qquad \Delta G^\circ_{\text{rxn1}} + \Delta\Delta G^\circ_{\text{1-2}} \tag{10}$$

$$P_2 + I_1 \rightarrow P_2 I_1 \qquad \Delta G^\circ_{\text{rxn2}} + \Delta\Delta G^\circ_{\text{2-1}} \tag{11}$$

Supplementary Equations (8) and (9) correspond to the intended SNOP hybridization reactions, and Supplementary Equations (10) and (11) correspond to the unintended side reactions between precursors and non-cognate probe instances.

In the ideal case, $\Delta G^\circ_{\text{rxn1}} = \Delta G^\circ_{\text{rxn2}}$, and $\Delta\Delta G^\circ_{\text{1-2}} = \Delta G^\circ_{\text{2-1}} = \infty$. In this situation, when the reactions are at equilibrium, SNOP product concentration normalization is perfect: There are no side reaction products P_1I_2 and P_2I_1, and when $[I_1] = [I_2]$, the intended products have the same concentration ($[P_1I_1] = [P_2I_2]$) regardless of any differences in the initial precursor concentration differences ($[P_1] \neq [P_2]$).

For a single nucleotide mismatch, $\Delta\Delta G^\circ$ typically ranges between 1 kcal/mol and 5 kcal/mol at the typical SNOP hybridization temperature of 60 °C. For $\Delta\Delta G^\circ = 3$ kcal/mol (typical 1 nt single-base mismatch), there will roughly 1% crosstalk. This means that SNOP can tolerate up to 100-fold difference in the concentrations of precursors P_1 and P_2 and still maintain product stoichiometry at roughly 3:1. Mathematically,

$$\frac{[P_1 I_2]}{[P_1 I_2] + [P_2 I_2]} \approx \frac{a/f}{1 + a/f} \tag{12}$$

where a is the concentration excess of P_1 over P_2, and $f \approx e^{\Delta\Delta G^\circ/RT}$ is the fold-enrichment due to $\Delta\Delta G^\circ$. When $a = f$, the fraction of I_2 bound to P_1 is roughly 50%, and because almost all I_1 is bound to P_1 due to the latter's concentration excess and favorable binding, the final ratio is approximately 3:1.

Differences in precursor concentrations can have a much larger effect when the $\Delta G^\circ_{\text{rxn}}$ values are different. For example, when $\Delta G^\circ_{\text{rxn1}} < \Delta G^\circ_{\text{rxn2}}$, the $P_1 + I_2 \rightarrow P_1 I_2$ reaction has standard free energy $\Delta G^\circ_{\text{rxn1}} + \Delta\Delta G^\circ_{\text{1-2}}$, compared to $P_2 + I_2 \rightarrow P_2 I_2$ with $\Delta G^\circ_{\text{rxn2}}$. The difference in the two energies is $\Delta\Delta G^\circ_{\text{1-2}} - (\Delta G^\circ_{\text{rxn2}} - \Delta G^\circ_{\text{rxn1}})$, smaller than $\Delta\Delta G^\circ_{1-2}$. This means that the fold-enrichment is smaller, and SNOP becomes less robust to increased concentrations of P_1. Likewise, if $\Delta G^\circ_{\text{rxn1}} > \Delta G^\circ_{\text{rxn2}}$, SNOP is less robust to increased concentrations of P_2. For this reason, uniformity of $\Delta G^\circ_{\text{rxn}}$ is desirable.

Supplementary Note 4: Time complexity of $\Delta G^\circ_{\text{rxn}}$ calculation and tag-oligo assignment. As mentioned previously, the reaction standard free energy can be calculated as the difference of the standard free energies of formation, based on published literature thermodynamics parameters [3]:

$$\Delta G^\circ_{\text{rxn}} = \Delta G^\circ(PI) - \Delta G^\circ(P) - \Delta G^\circ(I) \tag{13}$$

For the SNOP experiments described in this work, the length of the precursor is around 90 nt, and the length of the capture probe instance is 27 nt. The calculation of the partition function (ensemble) energy has time complexity $O(N^4)$, so calculation of $\Delta G^\circ(PI)$ is roughly 3 times more expensive than the calculation of $\Delta G^\circ(P)$. Furthermore, for a M-plex SNOP, there are only M distinct calculations of $\Delta G^\circ(P)$, but M^2 possible combinations for $\Delta G^\circ(PI)$. A comprehensive optimization of tag-oligo assignment is thus dominated by $\Delta G^\circ(PI)$ calculations and requires $O(M^2N^4)$ time, and is infeasible for moderately large values of M. With the randomized algorithm presented in Fig. 3 of the main text, the time complexity is $O(KN^4)$, where K is the number of iterations. Practically, K is chosen to be slightly bigger than M for a good tradeoff between runtime and performance.

To the extent that calculated values of $\Delta G^\circ(P)$, $\Delta G^\circ(I)$, and $\Delta G^\circ(PI)$ are imperfect and differ from true values, SNOP becomes less robust to variations in precursor concentrations. Our group's studies suggest that the standard error is around 1 kcal/mol for DNA oligos, so for a typical 3 kcal/mol single-base mismatch $\Delta\Delta G^\circ$, SNOP should be tolerant to concentration errors of up to 30-fold. Further improvements to DNA hybridization thermodynamics parameters can benefit SNOP tag-oligo assignment to improve product concentration uniformity.

Supplementary Discussion

SNOP Product Stoichiometry Analysis As mentioned in the main text, quantitation of relative concentrations of SNOP products was performed through normalization to a reference NGS library of the 64 desalted oligos at nominally identical concentrations. This is done to overcome the sequence bias of NGS. Supplementary Figure 13a and Supplementary Figure 14a show the total number of reads aligned to each product for the SNOP products and the desalted oligo library. There is roughly a 5-fold variation in reads even for the desalted library. Furthermore, the high reproducibility of reads across two independent experiments (Supplementary Figure 13b and Supplementary Figure 14b) indicate that the reads variation does correspond to systematic sequence-based bias, rather than random error.

Supplementary Figure 15a shows the inferred concentrations distribution of the 64-plex SNOP products, and the library of 64 individually PAGE-purified oligos at nominally identical concentrations. We next performed 64 individual digital PCR reactions with specific primers to each of the 64 SNOP products, in order to obtain an independent measure the concentrations (Supplementary Figure 15c). The inferred SNOP product concentration distribution is very similar to that from our NGS studies (Supplementary Figure 15d). Because of the longer protocol times associated with running many digital droplet PCR reactions, we did not perform similar quantitation on the 256-plex SNOP products.

Supplementary Figure 16a shows the NGS reads and reproducibility of 256-plex SNOP products. There is significantly higher NGS bias, with reads varying more than 50-fold for the 256 precursors at nominally identical concentrations. Here, we determined SNOP product concentrations by normalizing to precursor reads.

In the NGS library preparation process, we chose to use the ligation-based method because we believed it would be subject to less bias than a PCR-based method for appending sequencing primers. Supplementary Figure 17 confirms our understanding of the relative biases of ligation vs. PCR; whereas the SNOP product library constructed by ligation has only about a 4-fold high-to-low concentration variation, the PCR-generated library more than 50-fold variation.

Probe Instance Bias Probe instance bias refers to the potential differences in the concentrations of the different probe instances. Significant probe instance bias would limit the ability of SNOP to perform concentration normalization, and it is likely that the current SNOP product concentration distribution (Fig. 5a of main text) reflects probe instance bias.

To study the effects of probe instance bias, we ordered the randomer capture probe both as a machine mixed synthesis and as a "hand mixed" split-pool synthesis (Supplementary Figure 19a). In machine mixed synthesis, both nucleotide phosphoramidites are introduced simultaneously at equal concentrations, but different nucleotide incorporation efficiencies will result in probe instance bias. In contrast, for the hand mixed synthesis, roughly 1/2 of the oligos are physically separated to add the relevant nucleotide, and then the synthesis products are pooled; this occurs for each of the 6-8 degenerate nucleotides used in the SNOP capture probe.

Supplementary Figure 19b and Supplementary Figure 19c shows the concentration distribution of 64-plex and 256-plex SNOP, respectively. The hand mix capture probe synthesis clearly shows lower SNOP product concentration bias than the machine mix synthesis, supporting our understanding of the SNOP mechanism. Supplementary Figure 20 shows that the purity of SNOP products are not significantly affected by the the probe instance instance.

Scaling SNOP To verify that SNOP can be scaled to purification of larger quantities of oligonucleotides, we here show our workflow and experimental results for SNOP producing 1.5 nmol of total oligo products. This represents a 75-fold increase over the 20 pmol scale reactions that we used for most of the experiments in this manuscript. To economically facilitate the higher SNOP scale, we switched from streptavidin-functionalized magnetic beads to streptavidin-coated agarose resin beads (Thermo Fisher), which are roughly a factor of 20 less expensive than magnetic beads for the same biotin binding capacity. Supplementary Figure 21 shows that the 1.5 nmol scale SNOP performs similarly as the 20 pmol scale SNOP, in both purity distribution and stoichiometry distribution.

Purification of biotin-functionalized oligos through SNOP. Biotin-functionalized oligonucleotides are desirable for applications such as probes for hybrid-capture enrichment in NGS library preparation. The original SNOP workflow uses biotinylated capture probes and streptavidin-coated magnetic beads, so biotinylated oligos would be directly captured by the beads. To facilitate purification of biotinylated oligos, here we show a revised SNOP workflows that uses EDC chemistry, using amino-modified capture probe and carboxyl-modified magnetic beads. The precursors possess an internal biotin modification to the 3' of the tag sequence. Supplementary Figure 22 shows that this workflow is effective for purifying biotin-functionalized oligos.

Alternative biotin-functionalization configurations for the capture probe. Different biotin-functionalization chemistries for the capture probe are commercially available. To test if any of these would

have a noticeable difference on SNOP effectiveness, we tried 3 different biotin functionalization chemistries (Supplementary Figure 23). These appear to all yield similar SNOP performance.

Supplementary References

[1] Zuker, M. Mfold web server for nucleic acid folding and hybridization prediction. Nucleic acids research, 31(13), 3406-3415 (2003).

[2] Zadeh, J. N. *et al.* NUPACK: analysis and design of nucleic acid systems. Journal of computational chemistry, 32(1), 170-173 (2011).

[3] SantaLucia, J. Jr. & Hicks, D. The thermodynamics of DNA structural motifs. *Annu. Rev. Biophys. Biomol. Struct.* **33**, 415-440 (2004).

[4] Zhang, D.Y. & Winfree, E. Control of DNA Strand Displacement Kinetics Using Toehold Exchange. *J. Am. Chem. Soc.* **131(47),** 17303-17314 (2009).

[5] Langmead, B., & Salzberg, S. L. Fast gapped-read alignment with Bowtie 2. Nature methods, 9(4), 357-359 (2012).
