# Supplementary material for: Simultaneous and stoichiometric purification of hundreds of oligonucleotides
Source: Nat Commun. 2018 Jun 25;9:2467. doi: 10.1038/s41467-018-04870-w (PMC6018234; doi:10.1038/s41467-018-04870-w)
Supplement: Supplementary file 3 — Description of Additional Supplementary Files [file 41467_2018_4870_MOESM3_ESM.pdf]

Description of Additional Supplementary Files

File Name: Supplementary Data 1
Description: The Supplementary Data file is an Excel spreadsheet that contains the sequences of the oligonucleotides and primers used in this study. The spreadsheet contains 4 tabs, organized following the layout of the main text: The "Fluorescence gel" tab refers to the sequences used within the fluorescence studies (Fig. 2 of the main text). The "64 plex NGS" tab refers to the 64 plex sequences used in for NGS analysis of both purity and stoichiometry. The "Advance Stoichiometry NGS" tab refers to the sequences used for the experiment used in Fig. 5 of the main text. Finally, the "256 plex" tab refers to the 256 plex used in Fig. 4 and 5 of the main text.
